# A case of pediatric Monteggia fracture–dislocation with ipsilateral distal radius fracture

**DOI:** 10.1016/j.xrrt.2024.06.002

**Published:** 2024-06-16

**Authors:** Yuki Yoshida, Takeshi Ikegami

**Affiliations:** Department of Orthopaedic Surgery, Fussa Hospital, Tokyo, Japan

**Keywords:** Forearm, Monteggia injury, Pediatric injury, Physeal fracture, Ipsilateral fracture, Closed reduction

The Monteggia fracture–dislocation is a rare yet complex injury characterized by a fracture of the ulna and dislocation of the radial head.^4^ Monteggia fracture–dislocation with an ipsilateral distal radius fracture is even rarer. Although similar rare cases have been reported, the majority of them necessitated surgical intervention due to the inherent instability associated with a concomitant ipsilateral distal radius fracture in Monteggia fracture–dislocations. Here, we present a rare case of pediatric Monteggia fracture–dislocation with an ipsilateral distal radius fracture, successfully managed through conservative treatment, involving prompt diagnosis and closed reduction.

## Case report

**Figure 1:**
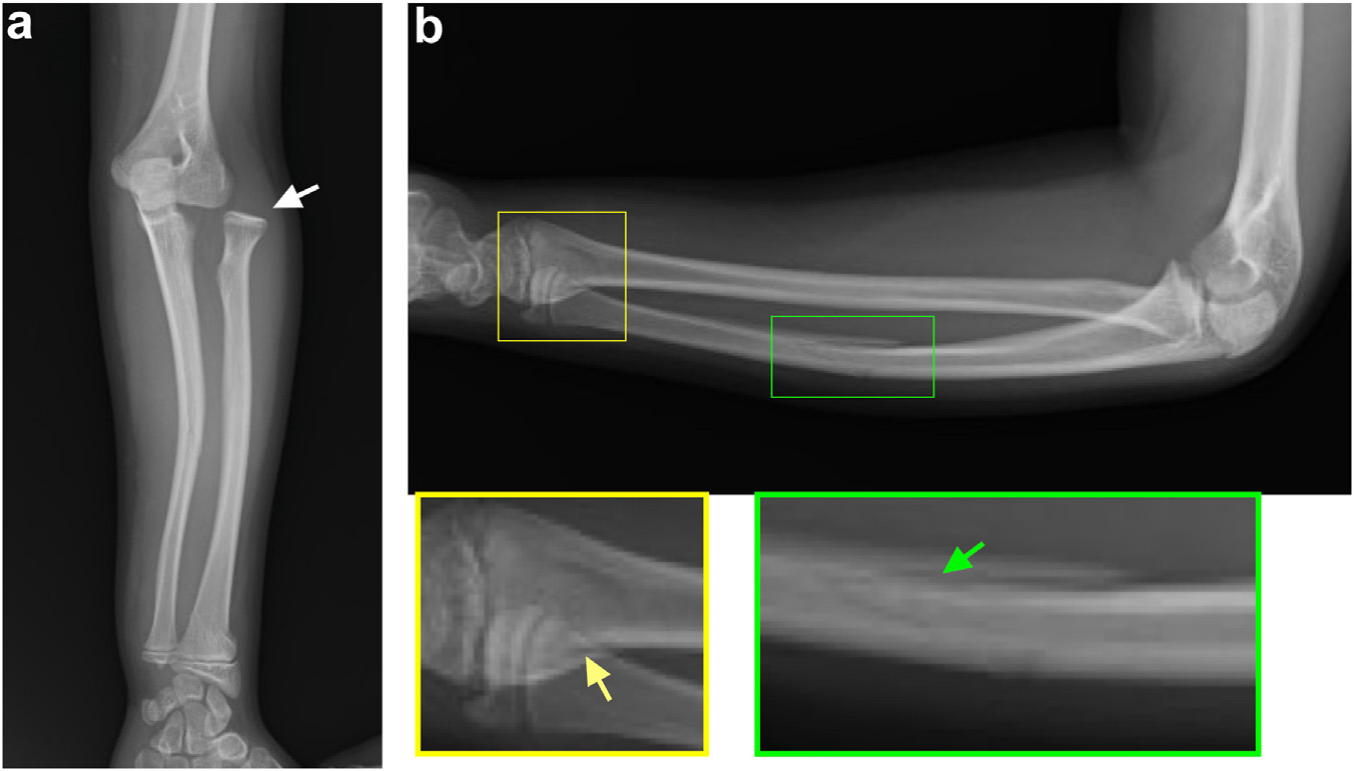
Initial radiographs. (**a**) Antero–posterior view displaying lateral dislocation of the radial head (*white arrow*). (**b**) Lateral view revealing minimally displaced fractures of the distal radius (*yellow arrow*) and ulna shaft (*green arrow*).

**Figure 2:**
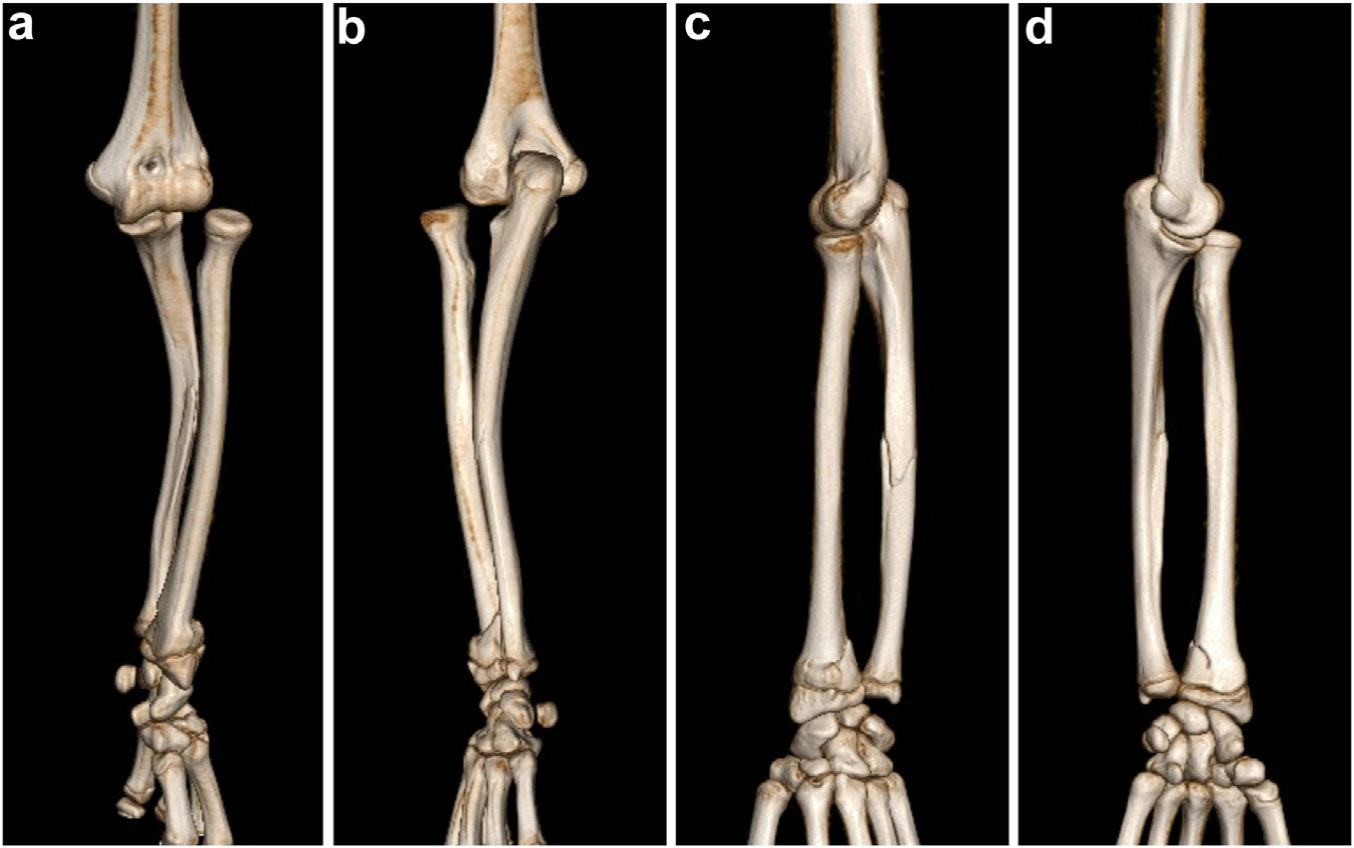
Three-dimensional CT images showing lateral dislocation of the radial head in the anterior and posterior view (**a**, **b**) and slightly anterior displacement in the lateral view (**c**, **d**). *CT*, computed tomography.

An 11-year-old girl was admitted to our hospital after falling while standing on a chair and outstretched her left hand. Her chief complaint was left elbow pain. The clinical assessment revealed tenderness and slight swelling in the left elbow joint, accompanied by a restricted range of motion. She also complained of mild wrist pain. There were no surface wounds or signs of neurovascular compromise. Radiographs showed a lateral dislocation of the radial head and minimally displaced fractures of the distal radius and ulna shaft (Fig. 1). Computed tomography revealed an anterolateral dislocation of the radial head with radial angulation of the ulna shaft fracture and a physeal fracture of the distal radius, indicative of a Bado type III Monteggia fracture–dislocation and a Salter-Harris type II fracture of the distal radius (Fig. 2).

**Figure 3:**
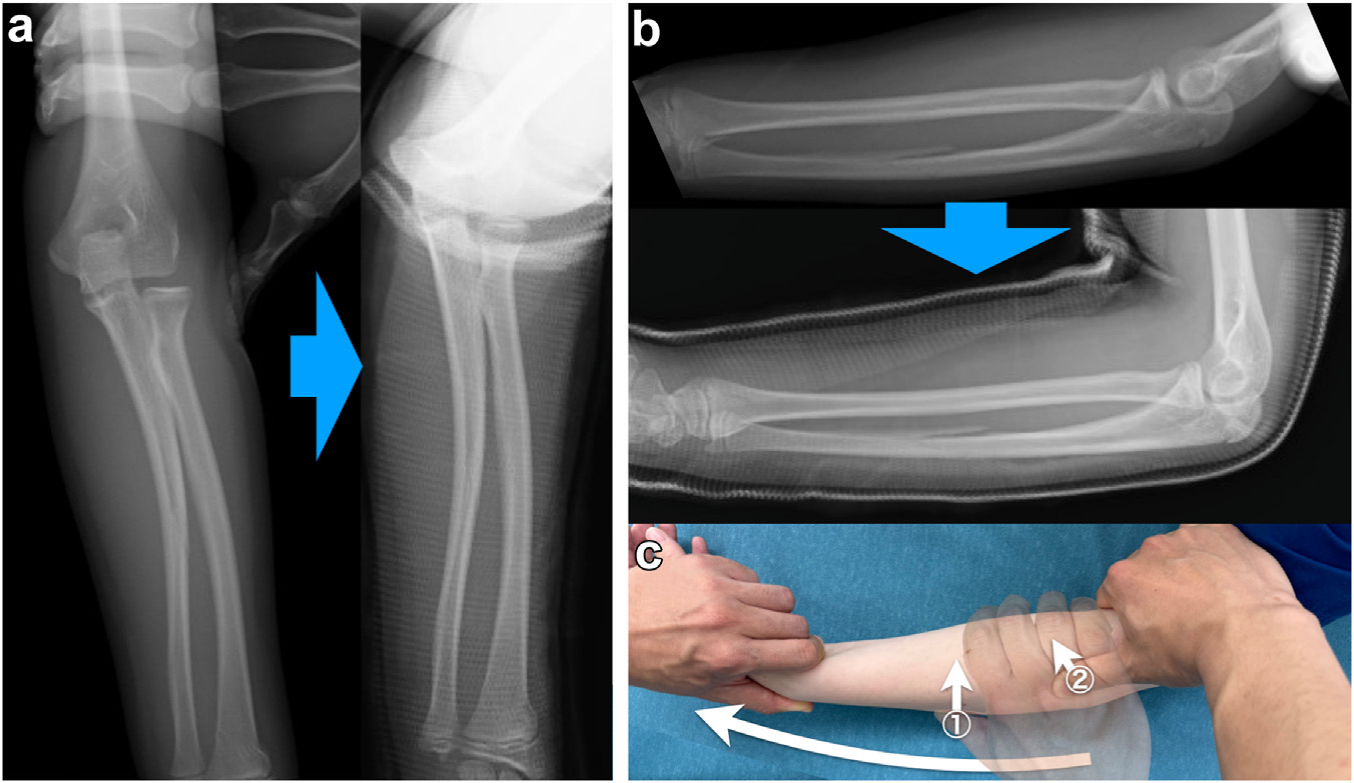
Fluoroscopic and radiographic images during closed reduction. (**a**) Antero–posterior view. (**b**) Lateral view. The radiograph after above-elbow cast was inaccurate and insufficient for evaluating the radial head dislocation. (**c**) Demonstration of the closed reduction. During forearm traction, the angulation of the ulna shaft was corrected by pushing the ulna from the posterior side (*white arrow 1*), followed by correcting the dislocation of the radial head by applying pressure from the lateral side (*white arrow 2*).

**Figure 4:**
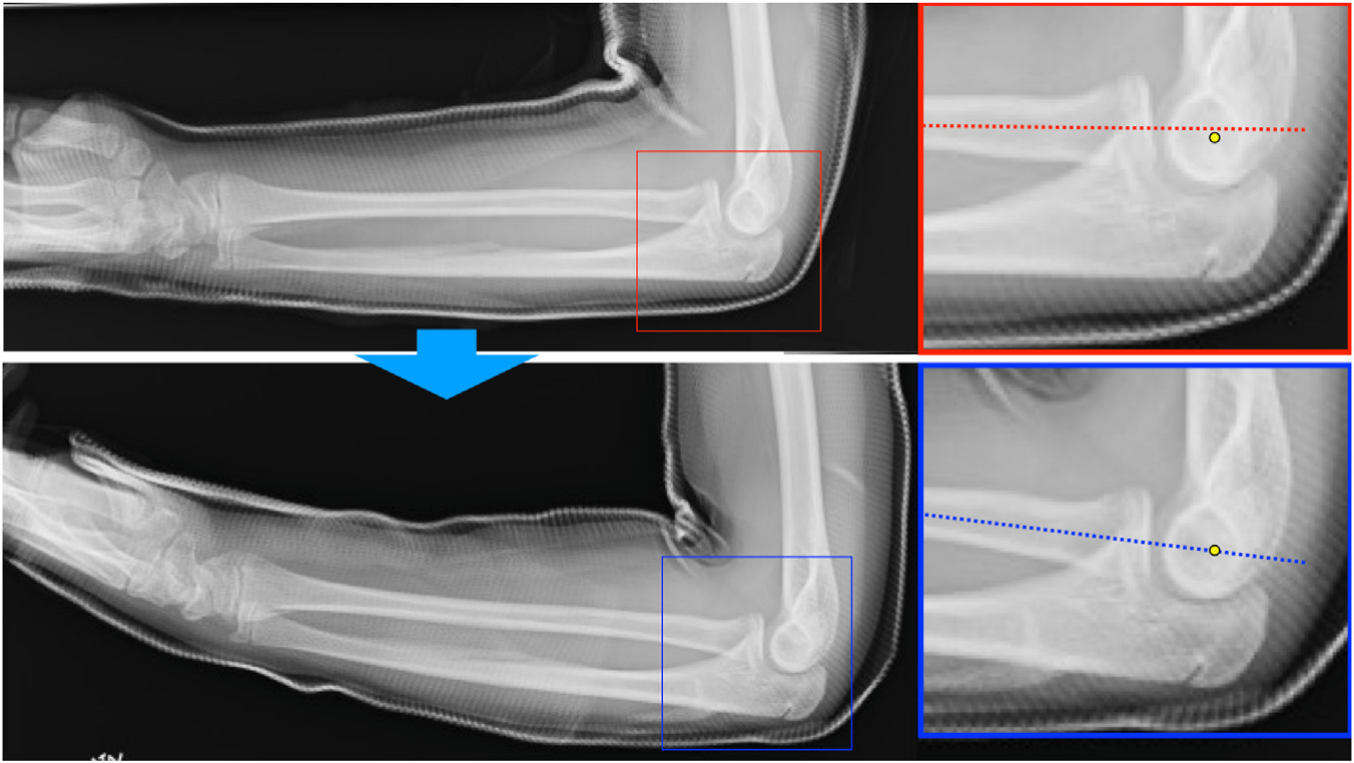
Radiographs were taken on the following day. A slight anterior displacement of the radial head was corrected with the elbow at 100° of flexion and the forearm fully supinated.

A closed reduction under axillary brachial plexus block was promptly performed on the day of the injury. Initially, the minimally displaced fractures of the distal radius and ulna shaft were manipulated with traction. As the radial fracture was slightly dorsiflexed, the wrist was gently flexed palmarly during traction. During this process, the angulation of the ulna shaft was corrected by pushing the ulna shaft from the posterior side. The dislocation of the radial head was then corrected by applying pressure to the radial head from the lateral side. Finally, the elbow was flexed to 90°, and the left upper limb was immobilized with an above-elbow cast in neutral rotation (Fig. 3). On the following day, she noticed a slight sensation of the radial head displacement. Although lateral displacement was managed by maintaining reduction through casting to hold the radial head, a slight anterior displacement of the radial head was revealed in the radiographs (Fig. 4). The radial head was rereduced by pushing it from the anterolateral side with the elbow in 100° of flexion and the forearm fully supinated. This position achieved and maintained reduction by tightening the interosseous membrane and relaxing the biceps tendon.^3^ Subsequently, conservative management was continued without recurrent dislocations.

**Figure 5:**
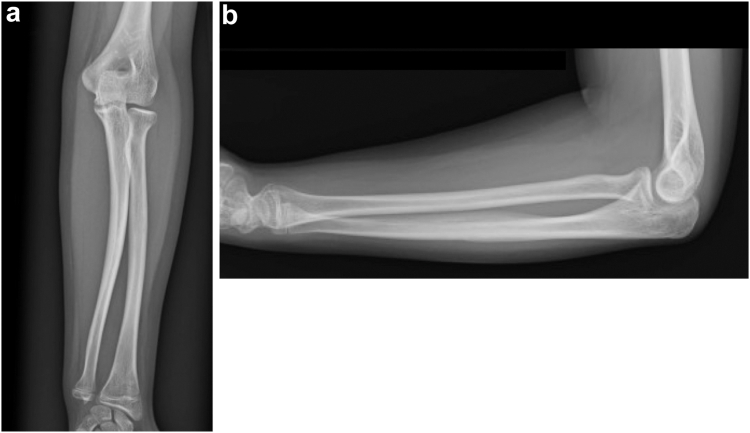
Three months postinjury. Radiographs showed complete healing of the radius and ulna fractures, with the radial head maintaining normal alignment. (**a**) Antero–posterior view. (**b**) Lateral view.

**Figure 6:**
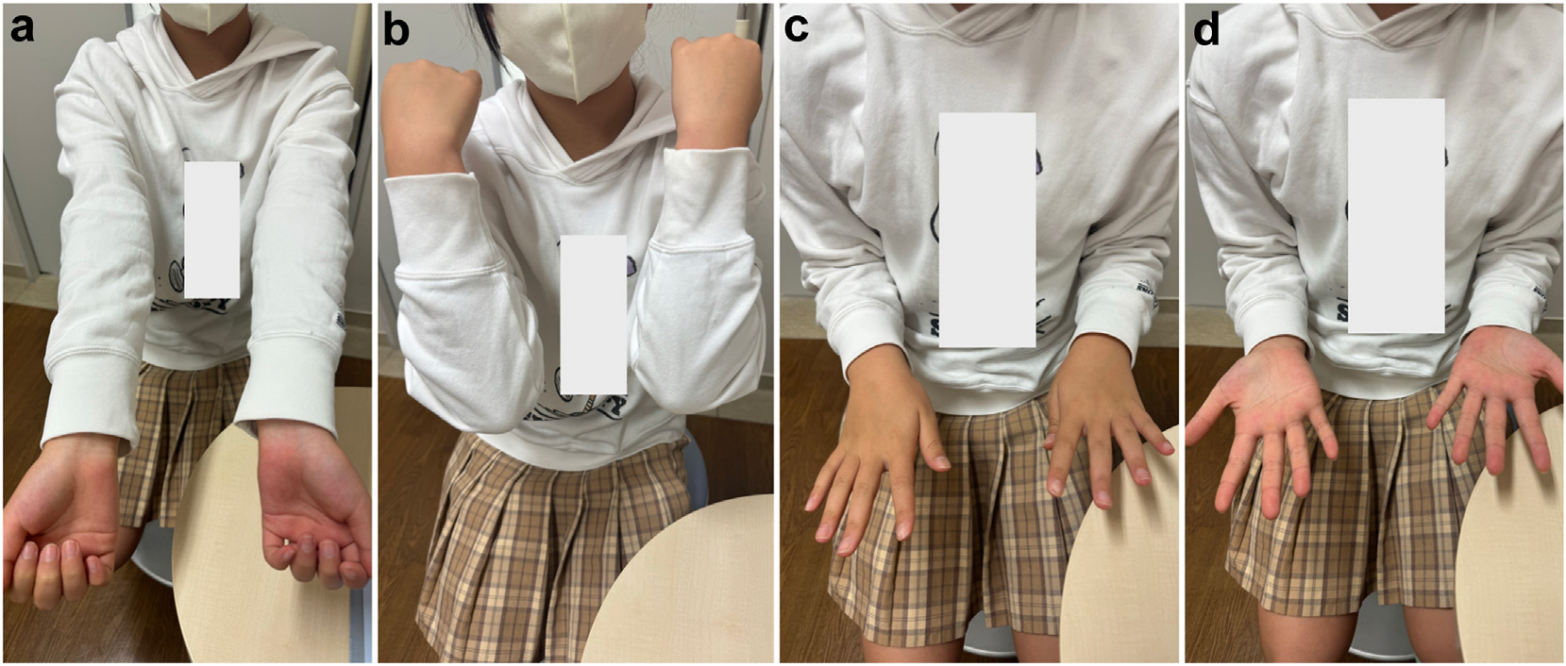
Range of motion at three months postinjury. (**a**) Elbow extension. (**b**) Elbow flexion. (**c**) Forearm supination. (**d**) Forearm pronation.

She was followed up weekly, confirming that the reduction was maintained. At 4 weeks postinjury, we transitioned from the cast to a removable splint, allowing elbow flexion and extension while keeping the forearm in the supinated position. At 6 weeks, bony union was confirmed without recurrent dislocations, leading to the removal of the splint and permitting pronation and supination. At 3 months, radiographs showed complete bony union with maintaining normal alignment (Fig. 5), and she demonstrated a full range of motion in the elbow and wrist joints with no observed complications (Fig. 6).

## Discussion

Pediatric Monteggia fracture–dislocation often occurs due to low-energy trauma, such as falls on the outstretched hand.^10^ It is imperative to sustain a heightened suspicion for radial head dislocation when ulna fractures are present to prevent oversight. This case represents a rare occurrence, involving not only Monteggia fracture–dislocation but also an ipsilateral distal radius fracture.

To the best of our knowledge, only nine cases of Monteggia fracture–dislocation with an ipsilateral distal radius fracture have been reported.^2^^,^^5^, ^6^, ^7^, ^8^, ^9^^,^^11^, ^12^, ^13^ All of these cases involved low-energy trauma same as typical pediatric Monteggia fracture–dislocation, resulting from low energy falls. Although low-energy trauma presents similarly to the typical pediatric Monteggia fracture–dislocation, cases involving a concomitant ipsilateral distal radius fracture tend to exhibit a higher prevalence of Bado type III rather than the more common type I observed in typical Monteggia fracture–dislocations.^12^ Six out of the ten cases, including this case were classified as Bado type III. The mechanism for Bado type III injury involves varus stress at the elbow combined with a fall on an outstretched hand, with the wrist dorsiflexed, resulting in both Monteggia fracture–dislocation and a distal radius fracture. It is hypothesized that varus stress on the elbow occurred during a fall from a chair, where the hand was supporting the impact, resulting in this injury.

In treating pediatric Monteggia fracture–dislocation, since these fractures are mostly incomplete greenstick fractures, performing reduction and maintaining it are relatively straightforward. Closed reductions of forearm fractures in children are preferred due to their potential for remodeling; as long as the growth plates are open, remodeling can take place. Therefore, conservative treatment is often viable in cases with a timely diagnosis.^1^ Due to the complexity of achieving closed reduction in Monteggia fracture–dislocation with an ipsilateral distal radius fracture, which creates instability from fractures in both the radius and ulna, surgical intervention has been commonly necessary in past cases.^2^^,^^5^, ^6^, ^7^, ^8^^,^^13^ Three out of the ten cases, including this case were conservative treatment with closed reduction.^9^^,^^11^ These three cases were relatively small displacement than other cases needed for surgical intervention. Although this case was promptly treated, there is acknowledgment of insufficient radiography evaluation after closed reduction. Despite the need for a second reduction the next day, favorable outcomes were achieved without recurrent dislocations. This emphasizes the importance of recognizing that Monteggia fracture–dislocation with an ipsilateral distal radius fracture is more unstable, and highlights the need for accurate radiography and frequent follow-up.^13^

If closed reduction proves unsuccessful, immediate operative intervention is necessary. Delays in treatment can lead to unfavorable long-term outcomes, including chronic valgus instability and radiocapitellar osteoarthrosis. Treatment options span from watchful waiting to operative measures, such as open reduction and stabilization of the radial head, annular ligament repair or reconstruction, or ulna and radius osteotomy. Given the challenging nature of chronic Monteggia injuries, accurate diagnosis and prompt treatment are crucial for achieving favorable treatment outcomes.^1^

## Conclusion

We presented a rare case of pediatric Monteggia fracture–dislocation with ipsilateral distal radius fracture. While the presence of a concomitant ipsilateral distal radius fracture typically adds instability to Monteggia fracture–dislocations, conservative treatment has demonstrated favorable outcomes with prompt diagnosis and closed reduction. Maintaining a heightened suspicion for associated injuries is imperative when dealing with forearm bone injuries. Conducting accurate radiography and implementing frequent follow-up are essential components of comprehensive care.

## Disclaimers:

Funding: No funding was disclosed by the authors.

Conflicts of interest: The authors, their immediate families, and any research foundation with which they are affiliated have not received any financial payments or other benefits from any commercial entity related to the subject of this article.

Patient consent: Obtained.
